# Assessment of citalopram and escitalopram on neuroblastoma cell lines: Cell toxicity and gene modulation

**DOI:** 10.18632/oncotarget.17050

**Published:** 2017-04-12

**Authors:** Laurent Sakka, Nathalie Delétage, Maryse Chalus, Youssef Aissouni, Valérie Sylvain-Vidal, Stéphane Gobron, Guillaume Coll

**Affiliations:** ^1^ Laboratoire d'Anatomie et d'Organogenèse, Laboratoire de Biophysique Sensorielle, NeuroDol, Faculté de Médecine, Université Clermont Auvergne, F-63000 Clermont-Ferrand, France; ^2^ Neuronax SAS, Biopôle Clermont-Limagne, F-63360 Saint-Beauzire, France; ^3^ Service de Neurochirurgie, Pole RMND, CHU de Clermont-Ferrand, Hôpital Gabriel-Montpied, 63003 Clermont-Ferrand Cedex, France; ^4^ Helixio, Biopôle Clermont-Limagne, F-63360 Saint-Beauzire, France; ^5^ Laboratoire de Pharmacologie Fondamentale et Clinique de la Douleur, NeuroDol, Faculté de Médecine, Université Clermont Auvergne, F-63000 Clermont-Ferrand, France

**Keywords:** neuroblastoma, citalopram, escitalopram, gene modulation, *MYCN*

## Abstract

Selective serotonin reuptake inhibitors (SSRI) are common antidepressants which cytotoxicity has been assessed in cancers notably colorectal carcinomas and glioma cell lines. We assessed and compared the cytotoxicity of 2 SSRI, citalopram and escitalopram, on neuroblastoma cell lines. The study was performed on 2 non-*MYCN* amplified cell lines (rat B104 and human SH-SY5Y) and 2 human *MYCN* amplified cell lines (IMR32 and Kelly). Citalopram and escitalopram showed concentration-dependent cytotoxicity on all cell lines. Citalopram was more cytotoxic than escitalopram. IMR32 was the most sensitive cell line. The absence of toxicity on human primary Schwann cells demonstrated the safety of both molecules for myelin. The mechanisms of cytotoxicity were explored using gene-expression profiles and quantitative real-time PCR (qPCR). Citalopram modulated 1 502 genes and escitalopram 1 164 genes with a fold change ≥ 2. 1 021 genes were modulated by both citalopram and escitalopram; 481 genes were regulated only by citalopram while 143 genes were regulated only by escitalopram. Citalopram modulated 69 pathways (KEGG) and escitalopram 42. Ten pathways were differently modulated by citalopram and escitalopram. Citalopram drastically decreased the expression of *MYBL2*, *BIRC5* and *BARD1* poor prognosis factors of neuroblastoma with fold-changes of -107 (p<2.26 10^−7^), -24.1 (p<5.6 10^−9^) and -17.7 (p<1.2 10^−7^). *CCNE1*, *AURKA*, *IGF*2, *MYCN* and *ERBB2* were more moderately down-regulated by both molecules. Glioma markers *E2F1*, *DAPK1* and *CCND1* were down-regulated. Citalopram displayed more powerful action with broader and distinct spectrum of action than escitalopram.

## INTRODUCTION

Experimental studies have demonstrated the promising properties of selective serotonin reuptake inhibitors (SSRI) such as citalopram in cancer diseases especially colorectal carcinoma [1, 2]. Citalopram and its S-eniantomer escitalopram have never been assessed and compared on neuroblastoma, the extracranial cancer the most commonly diagnosed during infancy [3]. Neuroblastoma is a developmental tumor which cell components originate in the neural crests and migrate to further contribute to the peripheral sympathetic nervous system: the sympathetic-chain ganglia trunks, the adrenal medulla and the prevertebral ganglia where neuroblastic proliferation may result in frequent tumor localizations.

Clinical presentation and prognosis of neuroblastoma display a surprising heterogeneity, ranging from benign tumors with cases of spontaneous regression to polymetastatic lethal disease. Genetic analysis of tumor samples performed in the last two decades correlated these prognostic disparities to an extensive series of genetic features actually considered as the prognosis markers of the disease notably *MYCN* [4, 5]. Those associated with a poor clinical outcome have become the potential targets for the development of new therapeutic approaches.

The aim of this work was to assess and compare the cytotoxicity of 2 SSRI, citalopram and escitalopram, on neuroblastoma cell lines including 2 non-*MYCN* amplified cell lines (rat B104 and human SH-SY5Y) and 2 human *MYCN* amplified cell lines (IMR32 and Kelly). The innocuity of citalopram and escitalopram on the myelin of the peripheral nervous system was assessed on primary human Schwann cells. Gene expression profiles of neuroblastoma prognosis markers using microarray method and quantitative real-time PCR (qPCR) analysis were determined to explore the molecular mechanisms of citalopram and escitalopram cytotoxicity on neuroblastoma cell lines.

## RESULTS

### Effects of citalopram and escitalopram on the viability of rat B104, human SH-SY5Y, IMR32 and Kelly neuroblastoma cell lines and human primary Schwann cells

Rat B104, human SH-SY5Y, IMR32 and Kelly neuroblastoma cells were exposed to increasing concentrations of citalopram and escitalopram. On all cell lines citalopram and escitalopram showed a concentration-dependent cytotoxicity, as assessed by the neutral red assay [6], but citalopram was more cytotoxic than escitalopram. In addition IMR32 was the cell line the most sensitive to both molecules. No toxicity was detected on human primary Schwann cells for citalopram or escitalopram.

**Figure 1:**
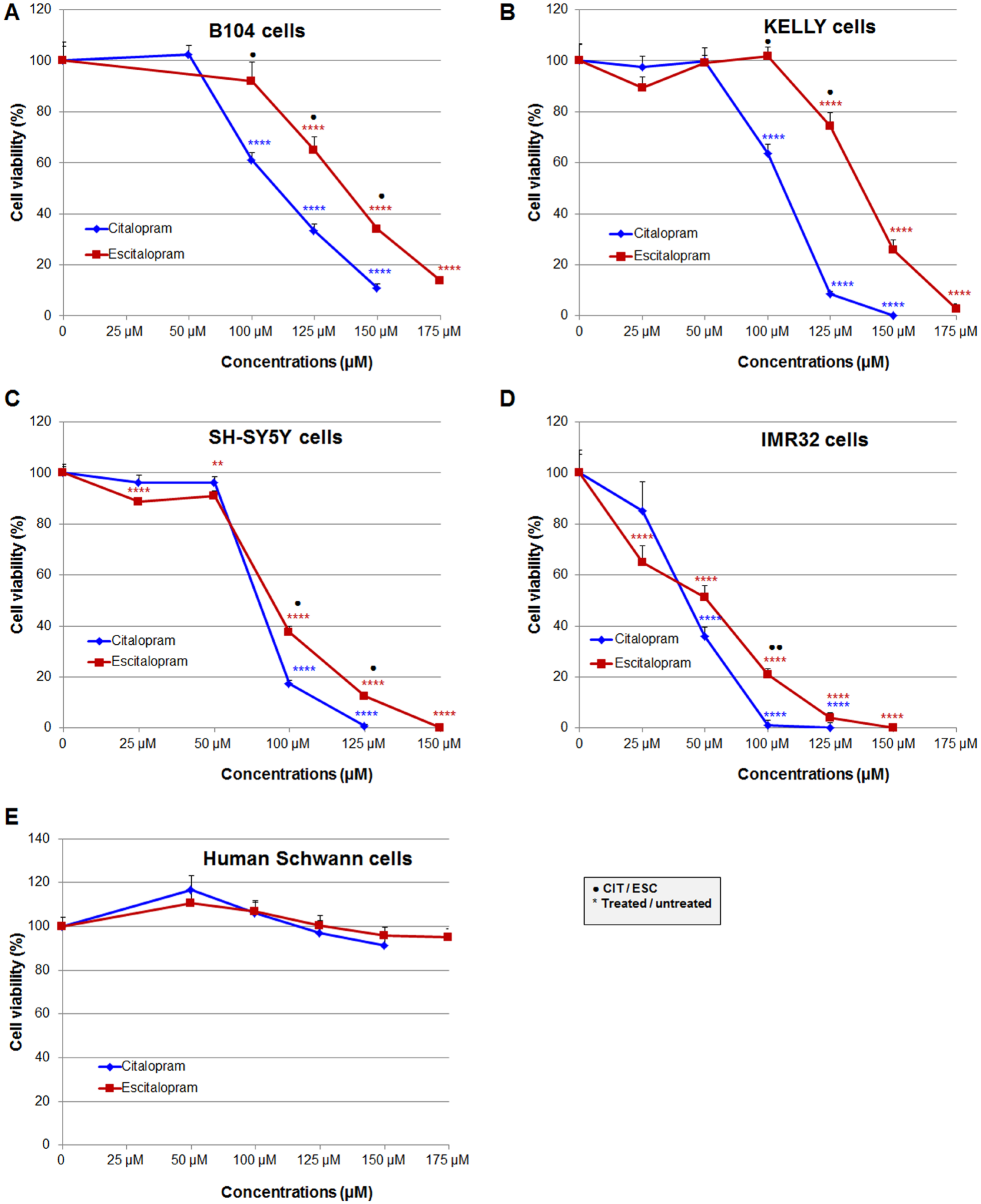
Comparative cytotoxicity of citalopram and escitalopram on rat B104, human Kelly, SH-SY5Y and IMR32 neuroblastoma cell lines and human Schwann cell primary cultures treated for 24 h with citalopram and escitalopram Cell viability was assessed using neutral red assay in **(A)** B104 cells, **(B)** Kelly cells, **(C)** SH-SY5Y cells, **(D)** IMR32 cells and **(E)** Human Schwann cells. Citalopram was more cytotoxic than escitalopram on all neuroblastoma cell lines. Human Schwann cells were resistant to citalopram and escitalopram treatment. Data represent treated cell viability as a percentage to untreated cells and are presented as mean values +/− SEM. Significantly different from untreated cells, ** p<0.01, **** p<0.001. Significant difference between citalopram and escitalopram treated cells, * p<0.05, ** p<0.01.

*B104 cell line*: Citalopram significantly decreased B104 cell viability, 61%, 33% and 11% at respectively 100, 125 and 150 μM. Escitalopram significantly decreased B104 cell viability, 65%, 34% and 14% at respectively 125, 150 and 175 μM (Figure 1A).

*Kelly cell line*: Citalopram significantly decreased Kelly cell viability, 64%, 9% and 0% at respectively 100, 125 and 150 μM. Escitalopram significantly decreased Kelly cell viability, 74%, 26% and 3% at respectively 125, 150 and 175 μM (Figure 1B).

*SH-SY5Y cell line*: Citalopram drastically decreased SH-SY5Y cell viability, 17%, 1% at respectively 100 and 125 μM. Escitalopram decreased cell viability to a lesser extent, 89%, 91%, 38% and 12% at respectively 25, 50, 100 and 125 μM; total mortality was observed at 150 μM (Figure 1C).

*IMR32 cell line*: Citalopram drastically decreased IMR32 cell viability, 36%, 1% and 0% at respectively 50, 100 and 125 μM. Escitalopram decreased cell viability to a lesser extent, 65%, 51%, 21%, 4% and 0% at respectively 25, 50, 100, 125 and 150 μM (Figure 1D).

*Primary human Schwann cells*: Citalopram or escitalopram did not impact human Schwann cell viability at 50, 100, 125, 150 and 175 μM (Figure 1E).

**Figure 2:**
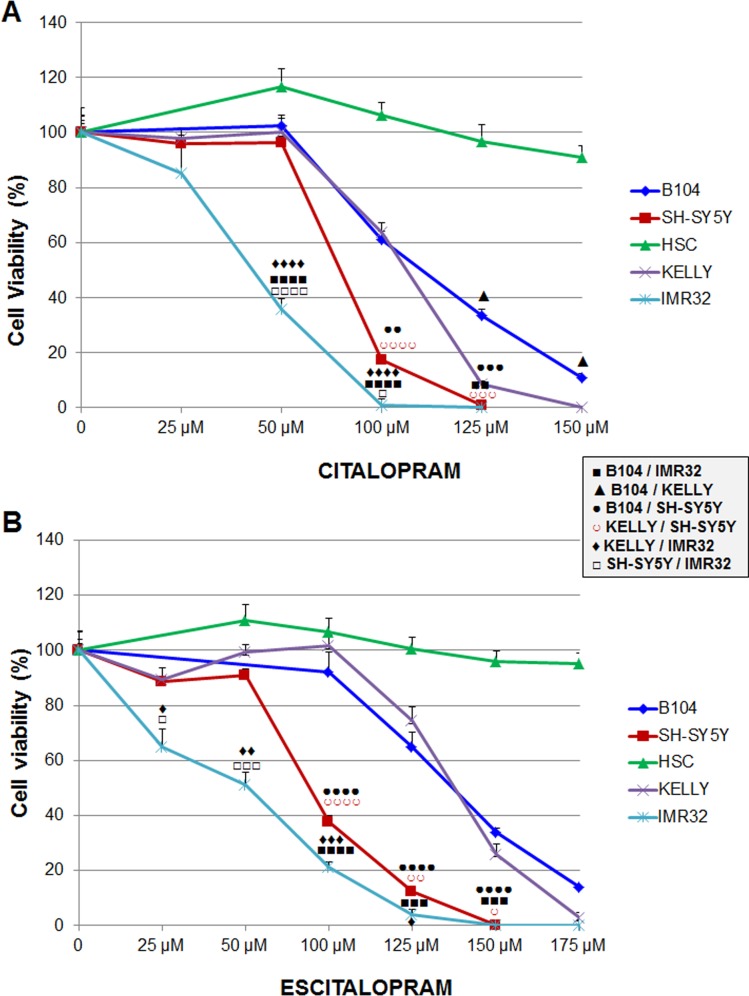
Sensitivity of B104, Kelly, SH-SY5Y and IMR32 neuroblastoma cell lines and human Schwann cells to citalopram or escitalopram **(A)** Viability after 24 h treatment with citalopram. Cytotoxicity of citalopram was dose-dependent; IMR32 were the most sensitive cells. No cytotoxic effect of citalopram was measured on human Schwann cells. **(B)** Viability after 24 h treatment with escitalopram. Escitalopram cytotoxicity was dose-dependent. IMR32 were the most sensitive cells. No cytotoxic effect of escitalopram was measured on human Schwann cells. Data represent treated cell viability as a percentage to untreated cells and are presented as mean values +/− SEM.

*Comparative sensitivity of neuroblastoma cell lines*: The sensitivity of neuroblastoma cell lines to citalopram and escitalopram are presented in Figure 2.

*After treatment with citalopram*: Briefly the viability of all cell lines was significantly decreased at concentrations of citalopram superior to 50 μM except IMR32 cells which viability was already altered at this concentration (Figure 2A). B104 cell line was the most resistant cell line. At lower concentrations Kelly cells provided a similar profile as B104 cells but were significantly more sensitive at higher concentrations. IMR32 was the most sensitive cell line with viability close to 0% at 100μM. SH-SY5Y displayed an intermediary profile with sensitivity between IMR32 and Kelly cells.

*After treatment with escitalopram*: IMR32 cells were the most sensitive cells with a viability that decreased from 25 μM of escitalopram. SH-SY5Y displayed a similar profile, with no significant difference with IMR32 at higher concentrations. The viability of B104 and Kelly cell lines was decreased at concentrations of escitalopram superior to 100 μM. Their profile was similar (Figure 2B).

### Effects of citalopram and escitalopram on gene expression

**Figure 3:**
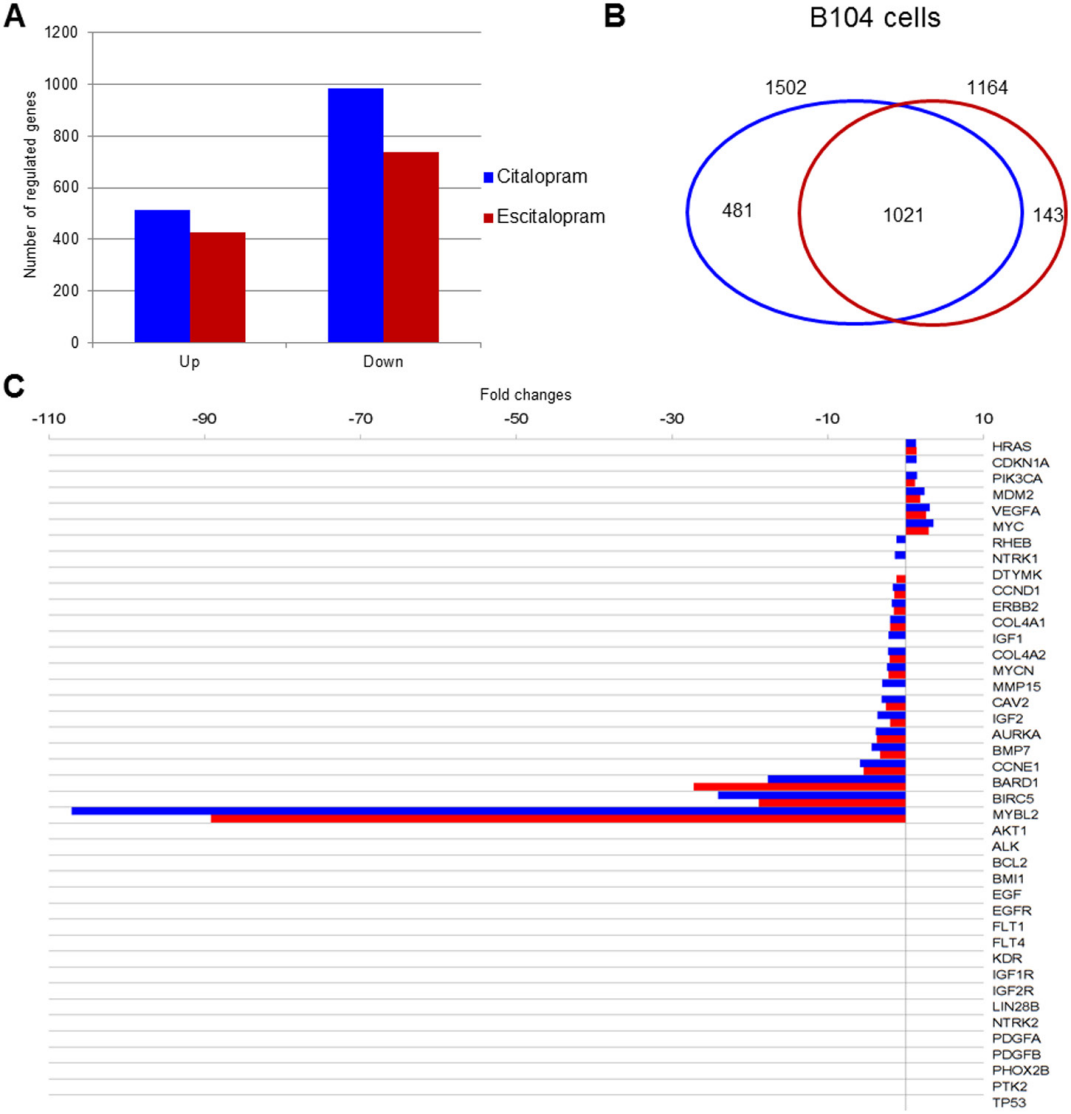
Modulation of gene expression by citalopram or escitalopram in B104 cells, Venn diagram, neuroblastoma prognostic marker gene expression **(A)** Modulation of gene expression by citalopram (blue) or escitalopram (red) in B104 cells. Histogram shows the number of up-regulated and down-regulated gene. The spectrum of action of citalopram is broader than escitalopram. **(B)** Venn diagram showing gene modulation by 24 h treatment with citalopram (blue) or escitalopram (red), fold change ≥ 2, and p<0.05. 1 196 genes are regulated by both molecules whereas 504 are specifically modulated by citalopram and 109 by escitalopram. **(C)** Neuroblastoma prognostic marker gene expression after treatment with citalopram (blue) or escitalopram (red). Prognosis markers are classified according to their fold change, with max p<7.36 10^−4^. The action of citalopram is more intense, its spectrum of action broader than escitalopram.

**Figure 4:**
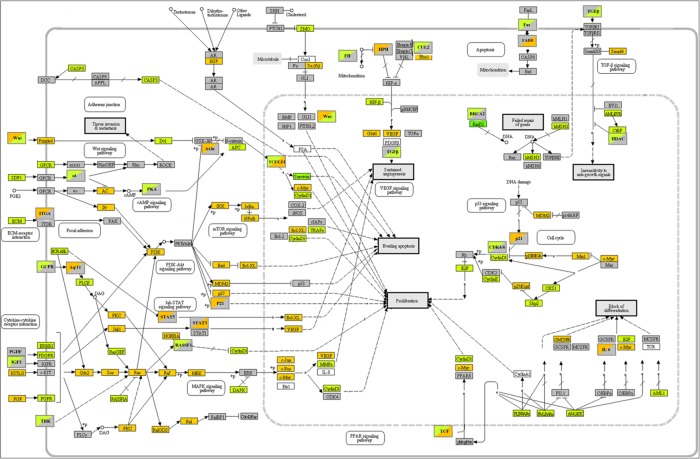
KEGG pathways in cancer B104 cells. Gene expression modulations by citalopram and escitalopram. Orange: up-regulation; green: down-regulation; grey: no regulation. Upper left corner: regulation by citalopram; lower right corner regulation by escitalopram.

*Microarray analysis*: Expression profile of B104 cells following a 24 h treatment with citalopram or escitalopram was studied by microarray experiments using 30 367 rat oligonucleotide probes. Citalopram and escitalopram modulated the expression of respectively 5 004 and 4 033 probes related to genes of known function. In addition, the expression of 3 979 probes was modulated by both citalopram and escitalopram. 1 586 probes were regulated only by citalopram while 461 probes were regulated only by escitalopram. More precisely, citalopram modulated the expression of 1 502 genes with a fold change ≥ 2, and p<0.05 including 516 up-regulated genes and 986 down regulated. Escitalopram modulated the expression of 1 164 genes with a fold change ≥ 2, and p<0.05. They included 428 up-regulated genes and 736 down-regulated genes. The expression of 1 021 genes with a fold change ≥ 2, and p<0.05 were modulated by both citalopram and escitalopram (Figure 3A, 3B). Genes differentially expressed following treatment of neuroblastoma cell line with citalopram or escitalopram were analyzed according to their pathways as recorded in the Kyoto Encyclopedia of Genes and Genomes (KEGG database). With p<10^−5^ citalopram modulated 69 pathways; the 4 most affected pathways were Cell Cycle (68 genes), Lysosome (60 genes), Pathways in Cancer (98 genes) and DNA Replication (28 genes) (Supplementary data 1). With the same p value, escitalopram modulated 42 pathways; the 4 most affected pathways were Cell Cycle (62 genes), Lysosome (56 genes), DNA Replication (29 genes) and Pathways in Cancer (78 genes) (Supplementary data 2). Ten signaling pathways were differently modulated by citalopram and escitalopram with p<6.83 10^−6^. Only 5 genes involved in Glioma pathway were differently modulated by citalopram and escitalopram (with p<0.04) (Supplementary data 3). The influence of citalopram and escitalopram on gene expression was first focused on genes that might be involved in the prognosis of neuroblastoma (Table 1, Figure 3C). The genes which expression was drastically inhibited were *MYBL2*, *BIRC5* and *BARD1* with a fold-change of respectively -107 (p<2.26 10^−7^), -24.1 (p<5.6 10^−9^) and -17.7 (p<1.2 10^−7^) after treatment with citalopram and respectively -89 (p<2.26 10^−7^), -18.8 (p<5.6 10^−9^) and -27.3 (p<1.2 10^−7^) after treatment with escitalopram. Gene expression of *CCNE1*, *AURKA*, *IGF*2, *MYCN* and *ERBB2* was significantly inhibited by both molecules whereas the expression of *IGF1* was inhibited only by citalopram. The expression of *MDM2* and V*EGFA* were significantly increased by both molecules. The expression of *AKT1*, *ALK*, *BCL*2, *IGF1R* and *IGF2R*, *EGF* and *EGFR*, *LIN28B*, *PDGFA* and *PDGFB*, *PHOX2*B, *TP53* was not modulated by either molecule. Several signaling pathways (Human Gene Database, GeneCards, PathCards) were more specifically altered by citalopram or escitalopram, notably PI3K-AKT, cell cycle, GPCR and MAPK signaling pathways. The study was extended to the expression of genes involved in general carcinogenesis (Table 2, Figure 4). Briefly, most genes were modulated by both molecules in the same way, 3 genes were modulated exclusively by escitalopram and 16 genes exclusively by citalopram. Particularly, *CCNA2* was drastically down-regulated by both citalopram and escitalopram with a fold-change of respectively -90 and -67 with p<4.86 10^−11^. The main signaling pathways modulated by both molecules were PI3K-AKT, GPCR, FGFR, MAPK and ERK. In the Glioma pathways (KEGG), 3 genes were down-regulated by both citalopram and escitalopram (*E2F1*, *DAPK1*, *CCND1*). Two genes (*CDK6* and *IGF1*) were down-regulated only by citalopram, while *CDKN1A* and *IL6* were up-regulated only by citalopram, and *STAT3* up-regulated only by escitalopram with p<10^−4^ (Table 1, Table 2).

**Table 1:** Neuroblastoma prognostic marker gene expression after treatment by citalopram or escitalopram

Gene symbol	Gene name	GenBank	CIT		ESC	
		Accession number	Fold change	p value	Fold change	p value
AKT1	V-Akt Murine Thymoma Viral Oncogene Homolog 1	**NM_033230**	-	**-**	-	**-**
ALK	Anaplastic Lymphoma Receptor Tyrosine Kinase	**NM_001169101**	-	**-**	-	**-**
AURKA	Aurora Kinase A	**NM_153296**	−3.87	5.97E-09	−3.72	5.97E-09
BARD1	BRCA1 Associated RING Domain 1	**NM_153296**	−17.71	1.20E-07	−27.25	1.20E-07
BCL2	B-Cell CLL/Lymphoma 2	**NM_016993**	-	**-**	-	**-**
BIRC5	Baculoviral IAP Repeat Containing 5 (survivin)	**NM_022274**	−24.09	5.63E-09	−18.88	5.63E-09
BMI1	BMI1 Proto-Oncogene, Polycomb Ring Finger	**NM_001107368**	-	**-**	-	**-**
BMP7	Bone Morphogenetic Protein 7	**NM_001191856**	−4.38	6.82E-08	−3.28	6.82E-08
CAV2	Caveolin 2	**NM_131914**	−3.07	7.70E-07	−2.53	7.70E-07
CCND1	Cyclin D1	**NM_171992**	−1.65	1.02E-04	−1.47	1.02E-04
CCNE1	Cyclin E1	**NM_001100821**	−5.91	9.27E-10	−5.39	9.27E-10
CDKN1A	Cyclin-Dependent Kinase Inhibitor 1A (p21)	**NM_080782**	1.38	7.36E-04	-	-
COL4A1	Collagen, Type IV, Alpha 1	**NM_001135009**	−2.02	4.40E-06	−2.03	4.40E-06
COL4A2	Collagen, Type IV, Alpha 2	**XM_001076134**	−2.29	7.03E-06	−2.10	7.03E-06
DTYMK	Deoxythymidylate Kinase	**NM_001106925**	-	**-**	-	**-**
EGF	Epidermal Growth Factor	**NM_012842**	-	**-**	-	**-**
EGFR	Epidermal Growth Factor Receptor	**NM_031507**	-	**-**	-	**-**
ERBB2	Erb-B2 Receptor Tyrosine Kinase 2	**NM_017003**	−1.78	5.41E-05	−1.52	5.41E-05
FLT1	Fms-Related Tyrosine Kinase 1 (VEGFR1)	**NM_019306**	-	**-**	-	**-**
FLT4	Fms-Related Tyrosine Kinase 4 (VEGFR3)	**NM_053652**	-	**-**	-	**-**
HRAS	Harvey Rat Sarcoma Viral Oncogene Homolog	**NM_001098241**	1.32	2.48E-05	1.38	2.48E-05
IGF1	Insulin-Like Growth Factor 1	**NM_001082479**	−2.2	3.64E-05	-	-
IGF2	Insulin-Like Growth Factor 2	**NM_031511**	−3.63	1.86E-04	−1.99	1.86E-04
IGF1R	Insulin-Like Growth Factor 1 Receptor	**NM_052807**	-	**-**	-	**-**
IGF2R	Insulin-Like Growth Factor 2 Receptor	**NM_012756**	-	**-**	-	**-**
KDR	Kinase Insert Domain Receptor (VEGFR2)	**NM_013062**	-	**-**	-	**-**
LIN28B	Lin-28 Homolog B (C. Elegans)	**XM_001069344**	-	**-**	-	**-**
MDM2	MDM2 Proto-Oncogene, E3 Ubiquitin Protein Ligase	**NM_001108099**	2.43	6.50E-07	1.88	6.50E-07
MMP15	Matrix Metallopeptidase 15	**NM_001106168**	−3.02	4.34E-05	-	
MYBL2	V-Myb Avian Myeloblastosis Viral Oncogene Homolog-Like 2	**NM_001106536**	−107.09	2.26E-07	−89.22	2.26E-07
MYC	V-Myc Avian Myelocytomatosis Viral Oncogene Homolog	**NM_012603**	3.53	7.44E-9	2.98	7.44E-09
MYCN	V-Myc Avian Myelocytomatosis Viral Oncogene Neuroblastoma Derived Homolog	**NM_001013096**	−2.43	2.08E-07	−2.19	2.08E-07
NTRK1	Neurotrophic Tyrosine Kinase, Receptor, Type 1	**NM_021589**	−1.38	3.49E-04	-	-
NTRK2	Neurotrophic Tyrosine Kinase, Receptor, Type 2	**NM_012731**	-	**-**	-	**-**
PDGFA	Platelet-Derived Growth Factor Alpha Polypeptide	**XM_006248919**	-	**-**	-	**-**
PDGFB	Platelet-Derived Growth Factor Beta Polypeptide	**NM_031524**	-	**-**	-	**-**
PHOX2B	Paired-Like Homeobox 2b	**NM_013158**	-	**-**	-	**-**
PIK3CA	Phosphatidylinositol-4,5-Bisphosphate 3-Kinase, Catalytic Subunit Alpha	**NM_133399**	1.43	1.43E-06	1.19	1.43E-06
PTK2	Protein Tyrosine Kinase (FAK)	**NM_013081**	-	**-**	-	**-**
RHEB	Ras Homolog Enriched In Brain	**NM_013216**	−1.20	8.35E-05	-	-
TP53	Tumor Protein P53	**NM_030989**	-	**-**	-	**-**
VEGFA	Vascular Endothelial Growth Factor A	**NM_001287107**	3.10	1.60E-05	2.61	1.60E-05

**Table 2:** List of genes involved in cancer pathways significantly modulated by citalopram or escitalopram treatment

Gene symbol	Gene name	GenBank	CIT		ESC	
		Accession number	Fold change	p value	Fold change	p value
ABL1	ABL Proto-Oncogene 1, Non-Receptor Tyrosine Kinase	**NM_001100850**	−1.65	6.71E-06	−1.89	1.59E-05
ADCY5	Adenylate Cyclase 5	**NM_022600**	1.44	7.36E-07	1.54	7.36E-07
APC2	Adenomatosis Polyposis Coli 2	**NM_001106769**	−8.28	8.77E-09	−5.44	8.77E-09
AXIN1	Axin 1	**NM_024405**	1.15	1.25E-03	-	-
BAD	BCL2-Associated Agonist Of Cell Death	**NM_022698**	1.23	9.43E-05	1.22	9.43E-05
BCL2L1	BCL2-Like 1 (bcl-xl)	**NM_001033670**	1.74	1.75E-06	1.44	1.75E-06
BRCA2	Breast Cancer 2, Early Onset	**NM_031542**	−6.70	5.78E-05	-	-
CASP3	Caspase 3, Apoptosis-Related Cysteine Peptidase	**NM_012922**	−1.38	1.51E-06	−1.29	1.51E-06
CCNA2	Cyclin A2	**NM_053702**	−89.96	4.86E-11	−67.41	4.86E-11
CCNE2	Cyclin E2	**NM_001108656**	−29.86	2.49E-06	−26.18	2.49E-06
CDK6	Cyclin-Dependent Kinase 6	**NM_001191861**	−2.97	6.37E-06	-	-
CDKN1B	Cyclin-Dependent Kinase Inhibitor 1B (P27, Kip1)	**NM_031762**	1.89	1.63E-06	1.72	1.63E-06
KS1B	CDC28 Protein Kinase Regulatory Subunit 1B	**NM_001135749**	−3.10	6.54E-09	−2.76	9.14E-08
CSF2RA	Colony Stimulating Factor 2 Receptor, Alpha, Low-Affinity(GMCSFR)	**NM_001037660**	1.57	4.23E-06	1.69	4.23E-06
CTBP2	C-Terminal Binding Protein 2	**NM_053335**	−1.57	5.21E-05	−1.50	5.21E-05
CUL2	Cullin 2	**NM_001108417**	−1.39	1.11E-04	-	-
CXCL12	Chemokine (C-X-C Motif) Ligand 12 (SDF1)	**NM_022177**	−3.60	1.87E-06	−3.04	1.87E-06
DAPK1	Death-Associated Protein Kinase 1	**NM_001107335**	−1.35	7.98E-04	−1.35	7.98E-04
DVL1	Dishevelled Segment Polarity Protein 1	**NM_031820**	−1.21	2.91E-04	−1.19	2.91E-04
E2F1	E2F Transcription Factor 1	**NM_001100778**	−3.08	8.78E-09	−2.91	8.78E-09
E2F8	E2F Transcription Factor 8	**XM_001080259**	−23.62	1.86E-08	−30.44	1.86E-09
EGLN2	Egl-9 Family Hypoxia-Inducible Factor 2(HPH)	**NM_001004083**	1.33	1.99E-03	-	-
FADD	Fas (TNFRSF6)-Associated Via Death Domain	**NM_152937**	-	-	2.17	6.59E-04
FAS	Fas Cell Surface Death Receptor	**NM_139194**	−6.46	1.85E-05	-	-
FGF9	Fibroblast Growth Factor 9	**NM_012952**	3.35	1.36E-05	4.17	1.36E-05
FGFR1	Fibroblast Growth Factor Receptor 1	**NM_024146**	−2.00	4.56E-05	−1.75	4.64E-05
FH	Fumarate Hydratase	**NM_017005**	−1.50	6.40E-06	-	-
FOS	Cellular Oncogene C-Fos	**NM_022197**	3.79	4.42E-05	3.01	4.42E-05
FZD1	Frizzled Class Receptor 1	**NM_021266**	−2.93	5.55E-08	−2.72	5.55E-05
GRB2	Growth Factor Receptor-Bound Protein 2	**NM_030846**	1.27	8.39E-05	1.29	8.39E-05
Hdac1	Histone Deacetylase 1	**NM_001025409**	−1.20	4.25E-04	-	-
IKBKB	Inhibitor Of Kappa Light Polypeptide Gene Enhancer In B-Cells, Kinase Beta	**NM_053355**	1.30	8.25E-05	1.14	8.25E-05
IL6	Interleukin 6	**NM_012589**	3.60	2.18E-04	-	-
ITGA3	Integrin, Alpha 3 (Antigen CD49C, Alpha 3 Subunit Of VLA-3 Receptor)	**XM_003750907**	1.33	2.89E-03	-	-
JAK1	Janus Kinase 1	**NM_053466**	1.47	2.70E-04	1.34	2.70E-04
JUN	Proto-Oncogene C-Jun	**NM_021835**	3.55	6.41E-05	3.19	6.41E-05
KITLG	KIT Ligand	**NM_021843**	3.15	3.01E-07	2.71	3.27E-04
LAMTOR1	Late Endosomal/Lysosomal Adaptor, MAPK And MTOR Activator 1	**NM_199102**	2.12	1.05E-06	2.06	1.05E-04
LAMTOR3	Late Endosomal/Lysosomal Adaptor, MAPK And MTOR Activator 3	**NM_001008375**	1.90	8.28E-07	1.77	8.28E-04
LAMTOR4	Late Endosomal/Lysosomal Adaptor, MAPK And MTOR Activator 4	**NM_001108330**	1.31	4.07E-05	1.36	4.07E-05
LAMTOR5	Late Endosomal/Lysosomal Adaptor, MAPK And MTOR Activator 5	**NM_001106462**	1.49	5.21E-05	1.50	5.21E-05
MAP2K1	Mitogen-Activated Protein Kinase Kinase 1	**NM_031643**	1.80	1.64E-05	1.51	1.64E-05
MMP2	Matrix Metallopeptidase 2	**NM_031054**	−6.66	2.37E-06	−5.04	6.65E-05
MSH2	MutS Homolog 2(hMSH2)	**NM_031058**	−2.82	3.95E-06	−2.55	3.95E-06
MSH3	MutS Homolog 3(hMSH3)	**NM_001191957**	−1.90	1.20E-06	−1.69	1.20E-06
NFKB2	Nuclear Factor Of Kappa Light Polypeptide Gene Enhancer In B-Cells 2 (P49/P100)	**NM_001008349**	1.39	1.25E-03	-	-
NFKBIA	Nuclear Factor Of Kappa Light Polypeptide Gene Enhancer In B-Cells Inhibitor, Alpha	**NM_001105720**	1.97	1.02E-06	2.42	1.02E-06
PDGFRL	Platelet-Derived Growth Factor Receptor-Like	**NM_001011921**	−2.83	3.03E-06	−2.80	3.03E-06
PRKCA	Protein Kinase C, Alpha	**NM_001105713**	1.65	6.24E-05	1.48	6.24E-05
PRKCD	Protein Kinase C, Delta	**NM_133307**	2.85	2.65E-09	2.69	2.65E-09
PRKDC	Protein Kinase, DNA-Activated, Catalytic Polypeptide	**XM_003751100**	−1.55	3.36E-04	-	-
RAD51	RAD51 Recombinase	**NM_001109204**	−49.86	6.06E-10	−41.01	1.74E-07
RAF1	Raf-1 Proto-Oncogene, Serine/Threonine Kinase	**NM_012639**	1.23	6.52E-05	1.26	6.52E-05
RALA	RAL	**NM_031093**	1.87	1.66E-05	1.72	1.66E-05
RALGDS	Ral Guanine Nucleotide Dissociation Stimulator	**NM_019250**	1.55	2.69E-04	1.39	2.69E-04
RASSF1	Ras Association (RalGDS/AF-6) Domain Family Member 1	**NM_001007754**	−1.67	3.57E-04	-	-
RBX1	Ring-Box 1, E3 Ubiquitin Protein Ligase	**NM_001034135**	1.21	1.29E-04	1.27	1.29E-04
RUNX1	Runt-Related Transcription Factor 1(AML1)	**NM_017325**	−1.40	1.64E-05	−1.35	1.64E-05
SKP2	S-Phase Kinase-Associated Protein 2, E3 Ubiquitin Protein Ligase	**NM_130413**	−3.73	4.44E-07	−4.58	4.44E-07
SLC2A1	Solute Carrier Family 2 (Facilitated Glucose Transporter), Member 1(Glut1)	**NM_138827**	2.23	2.72E-08	2.10	2.72E-08
SMAD4	SMAD Family Member 4	**NM_019275**	1.50	5.85E-06	1.40	5.85E-06
SMO	Smoothened, Frizzled Class Receptor	**NM_012807**	−1.88	3.16E-05	−1.36	3.16E-05
SOS2	Son Of Sevenless Homolog 2	**NM_001135561**	1.56	7.96E-04	1.35	6.40E-06
STAT3	Signal Transducer And Activator Of Transcription 3	**NM_012747**	-	-	1.40	1.15E-04
STAT5A	Signal Transducer And Activator Of Transcription 5A	**NM_017064**	1.39	2.96E-04	-	-
STAT5B	Signal Transducer And Activator Of Transcription 5B	**NM_022380**	1.97	2.42E-05	-	-
SUFU	Suppressor Of Fused Homolog	**NM_001024899**	1.41	3.80E-04	1.27	3.80E-04
TGFB2	Transforming Growth Factor, Beta 2	**NM_031131**	-	-	−2.23	2.49E-04
TRAF4	TNF Receptor-Associated Factor 4	**NM_001107017**	−2.12	8.60E-06	−2.00	8.60E-06
WNT1	Wingless-Type MMTV Integration Site Family, Member 1	**NM_001105714**	1.41	2.93E-03	-	-
WNT6	Wingless-Type MMTV Integration Site Family, Member 6	**NM_001108226**	−1.59	6.07E-03	−1.35	6.07E-05

### Gene expression analysis by qPCR using TaqMan low density array (TLDA)

Quantitative PCR studies were performed in B104, SH-SY5Y, Kelly and IMR32 cell lines to confront the microarray data obtained from B104 cell line. We specifically studied *MYCN*, *MYBL2*, *BIRC5*, *BARD1* and *AURKA*, poor prognosis markers of neuroblastoma, and *CCNA2* and *CCNE1* genes, involved in general carcinogenesis.

**Figure 5:**
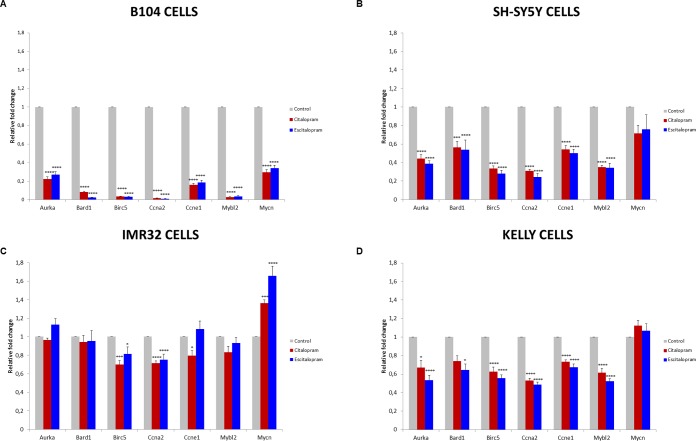
Fold change of representative genes after 24 h treatment with citalopram or escitalopram B104 cells **(A)**, SH-SY5Y cells **(B)**, IMR32 cells **(C)** and Kelly cells **(D)**. Duplicate values are normalized to 2 housekeeping gene expression and represented as fold induction compared with untreated cells set as 1. Results are expressed as means ± SEM, from 3 independent experiments (n=3). * p<0.05, *** p<0.005, ****p < 0.001.

*MYCN non-amplified cell lines (B104, SH-SY5Y)*: Citalopram and escitalopram strongly down-regulated *MYBL2*, *BIRC5*, *BARD1*, *AURKA, CCNA2* and *CCNE1* in B104 cells and at a lesser extent but significantly in SH-SY5Y cells. In B104 cells sharp down-regulation of *MYCN* was observed after treatment with citalopram or escitalopram, whereas in SH-SY5Y cells the down-regulation was a tendency. E2F1, involved in glioma pathways, was strongly down-regulated in B104 cells; its modulation was not explored in human cell lines (Figure 5A 5B, Table 3).

**Table 3:** Fold changes of specific genes after treatment by citalopram or escitalopram

Gene symbol	B104				SH-SY5Y				KELLY				IMR32			
	Citalopram		Escitalopram		Citalopram		Escitalopram		Citalopram		Escitalopram		Citalopram		Escitalopram	
AURKA	−4,48	****	−3,69	****	−2,27	****	−2,59	****	−1,49	*	−1,87	****	−1,03		1,13	
BARD1	−12,23	****	−40,08	****	−1,78	***	−1,86	****	−1,35		−1,56	*	−1,06		−1,05	
BIRC5	−28,34	****	−30,10	****	−2,97	****	−3,55	****	−1,60	****	−1,80	****	−1,43	***	−1,23	*
CCNA2	−63,59	****	−85,11	****	−3,23	****	−4,08	****	−1,88	****	−2,05	****	−1,40	****	−1,33	****
CCNE1	−6,20	****	−5,35	****	−1,84	****	−1,99	****	−1,37	****	−1,49	****	−1,26	*	1,08	
MYBL2	−35,32	****	−29,35	****	−2,86	****	−2,92	****	−1,63	****	−1,92	****	−1,20		−1,07	
MYCN	−3,37	****	−2,92	****	−1,40		−1,32		1,12		1,07		1,36	***	1,66	****
E2F1	−3,82	****	−3,60	****	ND		ND		ND		ND		ND		ND	

ND: not determined; * p<0.05, *** p<0.005, ****p < 0.001.

*MYCN amplified cell lines (Kelly, IMR32)*: In both cell lines *BIRC5* and *CCNA2* were significantly down-regulated by both molecules, the effect being more significant on Kelly than IMR32 cell line. The effect of citalopram tended to be more powerful on IMR32 than Kelly cell lines. *MYCN* was significantly up-regulated by escitalopram and at a lesser extent by citalopram in IMR32 cells, and not modulated by either molecules in Kelly cells. *AURKA*, *MYBL2* and *CCNE1* were significantly down-regulated by both molecules on Kelly cell line. *AURKA* and *MYBL2* were not affected by the treatment in IMR32 cell line. In this latter cell line, *CCNE1* was significantly down-regulated by citalopram only (Figure 5C, 5D, Table 3).

## DISCUSSION

Neuroblastoma is a developmental malignancy considered as the most frequent extra-axial solid tumor in childhood. It develops from neuroblasts of the neural crest that give birth to the peripheral sympathetic system through a multistage process of maturation involving successive expression of transcriptional factors. Disruption at discrete times of the process may be triggered by distinct oncogenes. Gradual identification of these oncogenes such as *MYCN* provides prognostic markers and therapeutic targets [5]. In the last two decades improvement has been performed in the therapeutic management of neuroblastoma but little has been gained in term of outcome for multi-metastatic patients. For these patients there is an urgent need to develop new therapeutic approaches.

SSRI are well known antidepressants which cytotoxic effects have been assessed with promising results in several cancer cell lines, more particularly colorectal carcinomas [1, 2]. They were initially suspected to promote cancer by historical studies which originally demonstrated serotonin promoted cell proliferation in the jejunum epithelium of rat [7] and in dimethylhydrazine-induced adenocarcinoma [8]. Later on, these data were contradicted by experiments showing the suppression of cell proliferation and tumor growth by fluoxetine and citalopram in two rodent models of colorectal carcinomas [1]. Then SSRI were found to promote fibrosarcomas, melanomas and mammary carcinomas on rodent models [9, 10] while positive association between SSRI usage and breast cancer risk was observed in epidemiological surveys [11, 12]. More recently case-control studies provided contradictory data, notably a reduced risk of colorectal cancer associated with SSRI use [13, 14]. Since, potent anticancer properties of SSRI have been more specified and extended to other types of cancers such as fluoxetine, paroxetine and citalopram on Burkitt lymphoma [15], fluoxetine on ovarian carcinoma [16], lung, colon, neuroblastoma, medulloblastoma, rhabdomyosarcoma, astrocytoma and breast cancer cell lines [17], and escitalopram on glioma C6 cells [18]. Nevertheless, potent carcinogenetic properties of citalopram have never been totally disclaimed. In mice exposed to recommended human dosages, citalopram induced significant DNA-strand breaking and micronuclei formation and displayed aneugenic and clastogenic effects on somatic cells, hence predisposing to the development of secondary tumors [19].

Up to now, citalopram and escitalopram have never been assessed in the indication of neuroblastoma and their potent efficacies have never been compared. We first assessed the 2 molecules on the rat B104 cell line. Our results led us to complement the study on human cell lines. Cell lines without amplification of *MYCN* (SH-SY5Y) or with amplification of *MYCN* (IMR32, Kelly) were selected to explore whether the cytotoxicity was still exerted on cells with *MYCN* amplification, a marker of poor prognosis present in 20% of patients [5].

In this work we demonstrate for the first time a significant cytotoxic effect of both citalopram and escitalopram in 4 neuroblastoma cell lines. We show a quantitative and qualitative difference of effect between the 2 molecules, citalopram showing a greater cytotoxicity than escitalopram on all neuroblastoma cell lines. The human IMR32 was the cell line significantly the most sensitive to both citalopram and escitalopram, while the lack of cytotoxicity on primary human Schwann cells suggested the innocuity of citalopram and escitalopram on the myelin sheath of the peripheral nervous system.

The most prominent feature of neuroblastoma resides in its clinical heterogeneity, with a prognosis ranging from cases of spontaneous regression in infants to extensive, metastatic, lethal disease [20]. Genetic profiling of human histological specimens has shown this clinical heterogeneity was related to a considerable genetic diversity [21]. Nevertheless, systematic correlation of genetic features to patient outcomes led to define prognostic markers [22] that might favor/inhibit neuroblastoma tumorigenesis by disturbing the equilibrium between cell survival and senescence signaling pathways and constitute potential therapeutic targets such as *MYCN* oncogene. Citalopram and escitalopram were cytotoxic on both *MYCN* and non-*MYCN* amplified cell lines and more notably on IMR32. Nevertheless our results suggest their action might not depend only on *MYCN* expression, since *MYCN* was not modulated in Kelly cells and increased in IMR32 cells, the cell line the most sensitive to both molecules.

To better understand the mechanisms of their cytotoxicity, we studied how gene expression was modified by citalopram and escitalopram using microarray method on B104 cells. The two molecules were compared at the same concentration of 100 μM because above 100 μM citalopram was too toxic to enable gene profiling (only 34% of cell viability was observed at 125 μM). Citalopram showed a broader spectrum of action than escitalopram on gene expression, modulating the expression of 1 502 genes whereas escitalopram modulated the expression of 1 164 genes with a fold change ≥ 2. The 2 molecules exerted distinct spectrums of action: 481 genes were regulated only by citalopram while 143 genes were regulated only by escitalopram.

Our study was focused on the genes identified as prognosis markers and potential therapeutic targets of neuroblastoma [4, 5, 21, 23, 24]. Concerning poor prognosis factors, when gene expression was modulated by both citalopram and escitalopram, the action was always a decrease except for *MDM2* and *VEGFA*. The action of citalopram was consistently more intense than escitalopram except for *HRAS*. The expression of 4 poor prognosis markers, *MYBL2*, *BIRC5*, *BARD1* and *AURKA* were drastically decreased by both molecules using the microarray analysis. These data were confirmed by the qPCR analysis in rat B104, human SH-SY5Y and Kelly cell lines. In IMR32 cells, only *BIRC5* was significantly down-regulated.

The sharp down-regulation of *MYBL2* (B-MYB) by citalopram and escitalopram, as shown by the microarray method on B104 cells were confirmed by qPCR on SH-SY5Y and Kelly cell lines. These data may be crucial since *MYBL2* over-expression, identified in advanced stages of neuroblastoma, confers a chemo-resistance to neuroblastoma cells [25, 26]. MYBL2 promotes cell proliferation and survival. Cell proliferation might be promoted in association with several factors involved in cell cycle pathways such as E2Fs and cyclin A2 [27, 28, 29]. Interestingly, *E2F1* was strongly down-regulated by citalopram and escitalopram in B104 cells according to the microarray and qPCR analyses. *CCNA2* was strongly down-regulated in all cell lines including IMR32 and Kelly overexpressing *MYCN*. The stimulation of cell survival by MYBL2 could be mediated by the activation of BCL2 [30, 31] which was not modulated by either molecule. MYBL2 and MYCN have been demonstrated to participate in a reciprocal regulatory loop [31, 32]. In microarray study of B104 cells *MYCN* was decreased at a lesser degree than *MYBL2* by citalopram and escitalopram, but the p value was strongly significant (2.08 10^−7^). These modulations on B104 cells were confirmed by qPCR. The loop involving MYBL2 and MYCN might constitute a therapeutic target particularly for the 20% of aggressive neuroblastomas where amplification of *MYCN* is observed [32]. Inhibitors of MYCN transcriptional function have been designed [21]. They include compounds termed MYRAs (for MYC pathway Response Agents) that selectively target MYCN pathway [34] and inhibitors of *MYCN* expression by the use of antisense oligonucleotides [35, 36]. Surprisingly, *TP53*, a direct transcriptional target of MYCN in neuroblastoma [37], was not modulated by citalopram and escitalopram. In fact, mutational inactivation of *TP53*, rarely observed in neuroblastoma, has been identified only in relapsing tumors [38]. *MDM2*, another transcriptional target of MYCN and poor prognosis factor of neuroblastoma [38] was slightly but significantly (p<6.5 10^−7^) increased by the 2 molecules whereas *CCNE1*, transcriptional target of MYCN identified in stage 4 neuroblastoma [40, 41], was strongly down-regulated by citalopram and escitalopram in B104, SH-SY5Y and Kelly cell lines and by citalopram in IMR32 cell line.

In our study, we demonstrate a significant down-regulation of *BIRC5* in the four neuroblastoma cell lines by qPCR analysis. Over-expression of *BIRC5* has been associated with aggressive forms of neuroblastoma [42] and other several types of cancer [43]. Anti-apoptotic protein BIRC5 was recently shown to shift neuroblastoma cells from oxidative phosphorylation to anaerobic glycolysis, increasing their resistance to chemotherapy [44]. Promising results obtained by BIRC5 inhibitors in pancreatic cancers with complete remission of liver metastases [45] make BIRC5 a potential target of chemotherapy in neuroblastoma.

*BARD1* was significantly down-regulated by citalopram and escitalopram in B104, SH-SY5Y and Kelly cell lines using qPCR analysis. Oncogenic action of BARD1 in neuroblastoma has been demonstrated to be exerted by stabilizing AURKA involved in aggressive neuroblastomas [46, 47]. *AURKA* was down-regulated by citalopram and escitalopram in B104, SH-SY5Y and Kelly cell lines using qPCR analysis. The kinase AURKA stabilizes MYCN [48, 49] and constitutes an interesting target for anticancer drug development. Inhibitors of AURKA are being assessed in clinical trials [50].

The analysis of signaling pathways involving the markers modulated by citalopram and escitalopram suggests the two enantiomer molecules have distinct biological properties. Citalopram has a broader spectrum of action than escitalopram. Some of their mechanisms of action might be distinct. Citalopram modulated 69 pathways and escitalopram 42 pathways. The 4 pathways most affected by the two molecules were identical but the number of genes modulated by citalopram was significantly greater than those modulated by escitalopram, 98 *versus* 78 in Pathways in Cancer. In addition, 10 signaling pathways were significantly differently modulated by citalopram and escitalopram. In the Pathway Glioma 5 genes were differently modulated by citalopram and escitalopram but only with a p-value of 0.04.

We analyzed the markers of glioma modulated by the molecules. Cytotoxicity of SSRI has been formerly studied *in vitro* on several models of glioma. In a glioma C6 cell line fluoxetine and paroxetine were previously proved to induce apoptosis [51]. On the same model, escitalopram was showed to induce apoptotic activity at roughly the same dosages as in our study [18]. The mechanisms underlying this cytotoxicity remain partially understood. Using our model of a neuroblastoma cell line exposed to citalopram or escitalopram, the study of gene expression involved in the KEGG Glioma pathways provided mixed results. Interestingly *E2F1* and the key component of the pRb/E2F1 pathway *CCND1* were down-regulated by both citalopram and escitalopram. While the position of E2F1 as a prognosis marker of glioma is debated [52, 53], abnormalities of the p16/pRb/E2F pathway with over-expression of E2F are present in most of gliomas and suggest E2F1 as a potential therapeutic target [54]. Interestingly, *CDK6* and *IGF1* were down-regulated only by citalopram. Up-regulation of CDK6 might be correlated with the malignancy of glioma, promoting both its proliferative and invasive capabilities [55, 56]. In addition CDK6 activates transcription factor E2F1/2 that might activate several drug-resistant genes which are up-regulated in glioma cells [57, 58]. These data have suggested CDK6 might constitute a therapeutic target for malignant gliomas. *In vitro*, inhibitors of CDK6 have been shown to enhance the sensitivity to chemotherapy on glioma cell lines [56]. They induce cell-cycle arrest and senescence in human neuroblastoma cell lines [59]. Two genes contributing to the malignant progression of various cancer types notably gliomas WHO grade II–IV, *IL6* and *STAT3*, were modulated by either molecules [60]. *IL6* was up-regulated by citalopram while *STAT3* was up-regulated by escitalopram. In human neuroblastoma cell lines STAT3 mediates the inhibition of apoptosis by IL-6 and the up-regulation of BIRC5 and BCL2L1 by IL-6 two actions probably involved in the drug resistance of some human neuroblastomas [61]. These data show mixed effects of citalopram and escitalopram on gliomas that should require gene profile exploration on relevant cell lines.

In this study we demonstrate a cytotoxic effect of citalopram and escitalopram on 4 neuroblastoma cell lines and their innocuity on primary human Schwann cells. The 2 molecules were active on both *MYCN* and non-*MYCN* amplified cell lines. The effect of citalopram was significantly more powerful than escitalopram on all neuroblastoma cell lines. To better understand their mechanisms of cytotoxicity, we studied the modification of gene expression induced by citalopram and escitalopram using microarray hybridization and qPCR. Citalopram was consistently more powerful on gene modulation with a broader spectrum of action on signaling pathways than escitalopram.

## MATERIALS AND METHODS

### Cytotoxicity assay

Cytotoxicity of citalopram and escitalopram was assessed on a rat B104 neuroblastoma cell line, SH-SY5Y, IMR32 and Kelly human neuroblastoma cell lines and a primary human Schwann cell culture (HSC).

*Chemical compounds*: Stock solutions of 10 mM citalopram (Sigma-Aldrich, Saint-Quentin-Fallavier, France; PubChem CID: 77995) and 50 mM escitalopram (Sigma-Aldrich, Saint-Quentin-Fallavier, France; PubChem CID: 146571) were prepared in distilled water. Dilutions of citalopram and escitalopram were made just prior to each experiment.

*Cell culture*: Rat B104 neuroblastoma cells (ICLC, Genova, Italy) and human SH-SY5Y neuroblastoma cells (ECACC, Salisbury, UK) were cultured in Dulbecco's Modified Eagle's Medium (DMEM) (Thermo Fisher Scientific, USA) supplemented with 2 mM glutamine, 100 U/ml penicillin, 100 μg/ml streptomycin and 10% fetal bovine serum (FBS) (Invitrogen) in a humidified atmosphere at 37°C with 5% CO_2_. Human IMR32 cells (ECACC, Salisbury, UK) were cultured in DMEM supplemented with 2 mM glutamine, 100 U/ml penicillin, 100 μg/ml streptomycin, 1% non-essential amino-acids (Invitrogen) and 10% FBS in a humidified atmosphere at 37°C with 5% CO_2_. Human Kelly cells (ECACC, Salisbury, UK) were cultured in RPMI (Invitrogen) supplemented with 2 mM glutamine, 100 U/ml penicillin, 100 μg/ml streptomycin and 10% fetal bovine serum (FBS) (Invitrogen) in a humidified atmosphere at 37°C with 5% CO_2_. HSCs isolated from human spinal nerves (ScienCell Research Laboratories, USA) were cultured on poly-L-lysine coated flasks in Schwann Cell Medium (ScienCell Research Laboratories, USA). Passage numbers were P5 for B104 cells, P19 for SH-SY5Y cells, P68 for IMR32 cells, P3 for Kelly cells and P3 for HSCs. The medium of all cell cultures was changed every 3 days.

*Cell treatment*: All neuroblastoma cell lines were plated onto poly-D-lysine-coated 96-well plates at a final density of 20 000 cells/well in complete medium. HSCs were plated onto poly-L-Lysine-coated plates at the same seeding density as neuroblastoma cells in Schwann Cell Medium. Treatment was administered 29 h after seeding. Five hours after seeding *B104 cells* were deprived of serum for 24 h and treated for 24 h with 100 μl/well of citalopram (50, 100, 125, 150 μM) or escitalopram (100, 125, 150, 175 μM) without serum. *SH-SY5Y cells* were treated for 24 h with 100 μl/well of citalopram (25, 50, 100, 125 μM) or escitalopram (25, 50, 100, 125, 150 μM) in presence of 3% FBS. *IMR32 cells* were treated for 24 h with 100 μl/well of citalopram (25, 50, 100, 125 μM) or escitalopram (25, 50, 100, 125, 150 μM) in presence of 3% FBS. *Kelly cells* were treated for 24 h with 100 μl/well of citalopram (25, 50, 100, 125, 150 μM) or escitalopram (25, 50, 100, 125, 150, 175 μM) in presence of 3% FBS. *HSCs* were treated for 24 h with 100 μl/well of citalopram (50, 100, 125, 150 μM) or escitalopram (50, 100, 125, 150, 175 μM) in Schwann Cell Medium.

*Neutral red assay*: Cell viability was evaluated using the neutral red uptake assay (Sigma-Aldrich, Saint-Quentin-Fallavier, France) after 24 h of treatment. The neutral red uptake assay is based on the ability of viable cells to incorporate and bind the neutral red dye within lysosomes [6]. Quadruplicate wells were used for each condition, and each experiment was repeated 3 to 4 times. Briefly, following exposure to treatments, cells were incubated for 2 h 30 with neutral red (40 μg/ml) dissolved in DMEM for B104, SH-SY5Y and IMR32 cells, in RPMI for Kelly cells and in Schwann Cell Medium for HSCs. After neutral red uptake cells were washed with Phosphate Buffered Saline (PBS). Incorporated dye was extracted from the cells by the addition of 150 μl/well of 1% acetic acid solution containing 50% ethanol. The plate was rapidly shaken for at least 10 min, or until the neutral red has been extracted from the cells. Absorbance was spectrophotometrically measured at 540 nm using the Multiskan Spectrum microplate reader (Thermo Scientific, Illkrich, France) with a background absorbance without cells as a blank. Cell viability of untreated cells was set to 100%. Treated cell viability was expressed as a ratio to untreated cell viability.

*Statistics*: Results were expressed as means ± SEM of 3-4 experiments. Statistical significance between sets of data was determined by ANOVA followed by the Dunnett post-hoc test. Differences were considered as statistically significant when p<0.05.

### RNA extraction

B104, SH-SY5Y, Kelly and IMR32 cells were seeded into 25 cm^2^ flasks at a density of 62 500 cells/cm^2^. B104 and Kelly cells were exposed to 100 μM citalopram or escitalopram for 24 h. IMR32 and SH-SY5Y were exposed to 75 μM citalopram or escitalopram for 24 h. Total RNA was extracted using the RNeasy Plus Mini Kit (Qiagen, Courtaboeuf, France) following the manufacturer's protocol. RNA quantity and purity (260/280 > 1.8) were determined using NanoDrop ND-1000 spectrophotometer (Thermo Scientific, Illkrich, France). RNA integrity was determined using Agilent 2100 Bioanalyzer and RNA 6000 Nano Kit (Agilent Technologies, Waldbronn, Germany). Only RNA samples with a RIN number > 8.0 were used for microarray analysis and qPCR analysis.

### Microarray analysis

Gene-expression profile was performed with RNA extracted from the B104 neuroblastoma cell line. Each experimental condition (treatment by citalopram, escitalopram and control) was performed in duplicates.

*Sample labeling and microarray hybridization*: Total RNA from B104 cells was reverse transcribed and the cRNA was labeled using Agilent Low Input Quick Amp Labeling Kit One-color (Agilent Technologies) following the manufacturer's protocol (One-color Microarray-Based Gene Expression Analysis v6.9). A hundred and fifty nanograms of each total RNA sample were used for linear T7-based amplification step and incorporation of cyanine 3-CTP for the production of Cy3-labeled cRNA. Labeled cRNA was purified using the RNeasy Mini kit (Qiagen) and quantified using NanoDrop ND-1000 spectrophotometer (Thermo Scientific, Illkrich, France). Only labeled cRNA samples with cRNA yields > 0.825 ng and specific activity > 6 pmol Cy3 per μg cRNA were selected for hybridization. Labeled cRNA quality (broad band size from 200 to 2000 nucleotides) was checked using Agilent 2100 Bioanalyzer with RNA 6000 Nano kit (Agilent Technologies, Waldbronn, Germany). Fragmentation and hybridization mixes were prepared using the Agilent Gene Expression Hybridization kit (Agilent Technologies). Subsequently, 600 ng Cy3-fragmented cRNA in hybridization buffer were hybridized 17 h at 65°C to SurePrint G3 Rat Gene Expression 8×60K microarray (Agilent Technologies Design ID 028279). Slides were washed with Agilent Gene Expression Wash Buffer I for 1 minute at room temperature followed by a second wash with Agilent Gene Expression Wash Buffer II for 1 minute at 37°C. Fluorescence signals of hybridized microarrays were detected at 3 μm resolution using the Microarray Scanner System G2505C (Agilent Technologies).

*Data collection and processing*: Raw data were extracted from scanned array images using Agilent Feature Extraction 11.5 software. Data were then analyzed using GeneSpring GX 12.0 software (Agilent Technologies). The raw signals were log transformed and normalized using percentile shift normalization method, the value was set at 75th percentile. For each probe, the median of the log summarized values from all the samples was calculated and subtracted from each of the samples to get transformed baseline. After normalization, probes were filtered according to quality flags set by the Agilent Feature Extraction software. Descriptive analysis was performed with GeneSpring GX 12.0: principal component analysis, box plots, unsupervised hierarchical clustering by Pearson's distance measure on average linkage. Gene significance was performed to determine differentially expressed genes through ANOVA (more than two groups) or unpaired t-test using the following parameters: p < 0.05, Benjamini-Hochberg false discovery rate as multiple testing correction, and SNK as a post-hoc test for ANOVA.

*Analysis of differentially expressed genes*: Bioinformatic analysis for enriched terms was performed using WEB-based Gene Set Analysis Toolkit (WebGestalt; http://www.webgestalt.org/) which performs a hypergeometric test for the enrichment of gene ontology (GO) terms and Kyoto Encyclopedia of Genes and Genomes (KEGG pathways) in the selected genes, followed by the Benjamini-Hochberg (BH) method for multiple test adjustment (adjP).

### Gene expression analysis by qPCR using TaqMan low density array (TLDA)

To confirm the results of microarray analyses we performed qPCR on RNA isolated from 3 independent cultures of untreated and citalopram or escitalopram-treated B104, SH-SY5Y, IMR32 and Kelly cells. Briefly, only RNA samples with a RIN number > 8.0 were used. From total RNA, 0.5 μg were reverse transcribed in a total volume of 20 μl using a High Capacity cDNA reverse transcription Kit (Life Technologies, Carlsbad, CA, USA). qPCR analysis was performed using custom designed arrays in the 384-well microfluidic cards TaqMan® Low Density Array (Life Technologies, Carlsbad, CA, USA). Each well contained specific, user-defined primers and 6-FAM (carboxyfluorescein) labeled TaqMan dihydrocyclopyrroloindole tripeptide Minor Groove Binder (MGB) probes to detect single genes. The card was configured into 3 identical sets of 23 genes including 2 housekeeping genes determined by TaqMan® Rat and Human Endogenous Control Arrays using geNorm algorithm: *PPIA* and *PGK1* for B104, *18S* and *POLR2A* for SH-SY5Y, *IPO8* and *POLR2A* for Kelly and *IPO8* and *18S* for IMR32 cells. Eight samples per card were processed in duplicate. A total of 100 μl reaction mixtures with 50 μl (100 ng) cDNA templates and an equal volume of TaqMan® universal master mix (Life Technologies, USA) was added to each line of microfluidic card after gentle vortex mixing. The array was centrifuged twice at 331 g for 1 min to distribute the samples from the loading port into each well. The card was then sealed and run on Applied Biosystems Prism 7900HT sequence detection system (Thermo Fisher Scientific, USA) according to recommended thermal cycling conditions. The threshold cycle (Ct) was automatically given by SDS v2.2 software package (Thermo Fisher Scientific, USA). Relative quantities (RQ) were determined using the equation: RQ = 2^−ΔΔCt^. Expression levels of genes of interest were normalized to the average expression level of housekeeping genes from the same sample. Gene relative expression was defined as the ratio of gene expression to that of the housekeeping genes. Fold changes were defined as the ratio of gene relative expression in treated cells to that in untreated cells. The data presented are given as the mean of the fold changes (n=3 per treatment) ± standard error mean (SEM). Statistical significance between sets of data was determined by ANOVA followed by the Dunnett post-hoc test. Differences were considered as statistically significant when p<0.05.

## SUPPLEMENTARY MATERIALS AND FIGURES




