# Supplementary material for: Assessment of citalopram and escitalopram on neuroblastoma cell lines: Cell toxicity and gene modulation
Source: Oncotarget. 2017 Apr 12;8(26):42789–807. doi: 10.18632/oncotarget.17050 (PMC5522106; doi:10.18632/oncotarget.17050)
Supplement: Supplementary file 1 [file oncotarget-08-42789-s001.pdf]

Assessment of citalopram and escitalopram on neuroblastoma cell lines: Cell toxicity and gene modulation

SUPPLEMENTARY MATERIALS

SUPPLEMENTARY DATA

Supplementary data 1: List of Kyoto Encyclopedia of Genes and Genomes (KEGG) pathways significantly modulated by 100 µM citalopram treatment

See Supplementary File 1

Supplementary data 2: List of KEGG pathways significantly modulated by 100 µM escitalopram treatment

KEGG pathway name	Number of differentially expressed genes	Number of genes in the pathway	p value
Cell cycle	62	124	2.44e-30
Lysosome	56	124	1.18e-24
DNA replication	29	36	6.76e-23
Pathways in cancer	78	319	4.41e-15
MAPK signaling pathway	67	269	1.94e-13
Endocytosis	59	230	1.70e-12
Nucleotide excision repair	23	44	5.04e-12
Ubiquitin mediated proteolysis	41	133	1.38e-11
Focal adhesion	50	186	1.41e-11
Glycolysis / Gluconeogenesis	30	78	2.33e-11
RNA transport	44	156	4.63e-11
Mismatch repair	15	22	2.45e-10
Pyrimidine metabolism	32	97	4.01e-10
Protein processing in endoplasmic reticulum	43	164	8.93e-10
Collecting duct acid secretion	16	27	9.49e-10
Alzheimer's disease	50	212	1.48e-09
Oocyte meiosis	34	115	2.22e-09
Pyruvate metabolism	19	41	3.93e-09
Neurotrophin signaling pathway	35	129	1.42e-08
Progesterone-mediated oocyte maturation	27	86	3.18e-08
Phagosome	42	185	1.11e-07
PPAR signaling pathway	24	76	1.88e-07
Rheumatoid arthritis	25	82	2.04e-07
Oxidative phosphorylation	37	156	2.08e-07
Valine, leucine and isoleucine degradation	19	51	2.38e-07
Purine metabolism	38	165	2.92e-07
Prostate cancer	25	87	6.43e-07
Axon guidance	31	126	9.75e-07
Aminoacyl-tRNA biosynthesis	20	61	1.06e-06
Fatty acid metabolism	17	46	1.18e-06
Small cell lung cancer	24	84	1.18e-06
Sphingolipid metabolism	16	42	1.61e-06
p53 signaling pathway	22	74	1.61e-06
Wnt signaling pathway	34	152	2.38e-06
Base excision repair	16	44	3.10e-06
Acute myeloid leukemia	18	55	3.68e-06
Chronic myeloid leukemia	21	73	4.85e-06
ErbB signaling pathway	23	85	4.85e-06
Toxoplasmosis	30	130	4.87e-06
ECM-receptor interaction	21	74	5.78e-06
Amino sugar and nucleotide sugar metabolism	16	47	7.43e-06
Hepatitis C	29	127	8.56e-06

Supplementary data 3: List of KEGG pathways significantly and differently modulated upon citalopram treatment *versus* escitalopram treatment

KEGG pathway name	Number of differentially expressed genes	Number of genes in the pathway	p value
Valine, leucine and isoleucine degradation	15	51	1.55e-10
Glycolysis / Gluconeogenesis	14	78	6.70e-07
Terpenoid backbone biosynthesis	7	14	7.62e-07
Fatty acid metabolism	11	46	7.62e-07
Butanoate metabolism	9	30	1.24e-06
Hypertrophic cardiomyopathy	13	80	2.85e-06
Peroxisome	13	80	2.85e-06
Steroid biosynthesis	7	19	5.54e-06
Proximal tubule bicarbonate reclamation	7	20	6.83e-06
Dilated cardiomyopathy	13	88	6.83e-06
Glioma	5	62	4.27e-02
