# Supplementary material for: Assessment of citalopram and escitalopram on neuroblastoma cell lines: Cell toxicity and gene modulation
Source: Oncotarget. 2017 Apr 12;8(26):42789–807. doi: 10.18632/oncotarget.17050 (PMC5522106; doi:10.18632/oncotarget.17050)
Supplement: Supplementary file 2 [file oncotarget-08-42789-s002.doc]

**Supplementary data 1: List of Kyoto Encyclopedia of Genes and Genomes (KEGG) pathways significantly modulated by 100 µM citalopram treatment**

| **KEGG pathway name** | **Number of**  **differentially expressed genes** | **Number of genes in the pathway** | **p value** |
| --- | --- | --- | --- |
| Cell cycle | 68 | 124 | 8.16e-31 |
| Lysosome | 60 | 124 | 1.85e-23 |
| Pathways in cancer | 98 | 319 | 5.83e-20 |
| DNA replication | 28 | 36 | 7.66e-19 |
| Focal adhesion | 67 | 186 | 8.67e-18 |
| MAPK signaling pathway | 83 | 269 | 3.05e-17 |
| RNA transport | 57 | 156 | 1.40e-15 |
| Oocyte meiosis | 47 | 115 | 4.07e-15 |
| Ribosome | 47 | 122 | 5.61e-14 |
| Endocytosis | 69 | 230 | 7.57e-14 |
| Ubiquitin mediated proteolysis | 49 | 133 | 9.61e-14 |
| Protein processing in endoplasmic reticulum | 54 | 164 | 8.76e-13 |
| Nucleotide excision repair | 25 | 44 | 2.84e-12 |
| Glycolysis / Gluconeogenesis | 34 | 78 | 3.70e-12 |
| Mismatch repair | 17 | 22 | 1.05e-11 |
| Insulin signaling pathway | 45 | 131 | 1.52e-11 |
| Progesterone-mediated oocyte maturation | 35 | 86 | 1.60e-11 |
| ECM-receptor interaction | 31 | 74 | 1.16e-10 |
| Pyrimidine metabolism | 36 | 97 | 1.73e-10 |
| Alzheimer's disease | 58 | 212 | 3.82e-10 |
| Neurotrophin signaling pathway | 42 | 129 | 4.68e-10 |
| Fatty acid metabolism | 23 | 46 | 5.30e-10 |
| p53 signaling pathway | 30 | 74 | 5.30e-10 |
| Phagosome | 52 | 185 | 1.10e-09 |
| Pyruvate metabolism | 21 | 41 | 1.83e-09 |
| Regulation of actin cytoskeleton | 55 | 208 | 3.76e-09 |
| Arrhythmogenic right ventricular cardiomyopathy | 28 | 71 | 4.20e-09 |
| GnRH signaling pathway | 34 | 99 | 4.84e-09 |
| Glutathione metabolism | 24 | 55 | 5.66e-09 |
| Spliceosome | 41 | 135 | 6.12e-09 |
| Hypertrophic cardiomyopathy | 29 | 80 | 1.86e-08 |
| ErbB signaling pathway | 30 | 85 | 2.02e-08 |
| Wnt signaling pathway | 43 | 152 | 2.49e-08 |
| Axon guidance | 38 | 126 | 2.73e-08 |
| Valine, leucine and isoleucine degradation | 22 | 51 | 3.10e-08 |
| Chronic myeloid leukemia | 27 | 73 | 3.38e-08 |
| Purine metabolism | 45 | 165 | 3.45e-08 |
| Small cell lung cancer | 29 | 84 | 5.54e-08 |
| Amoebiasis | 32 | 99 | 5.99e-08 |
| Collecting duct acid secretion | 15 | 27 | 1.05e-07 |
| RNA degradation | 27 | 77 | 1.12e-07 |
| Prostate cancer | 29 | 87 | 1.23e-07 |
| Dilated cardiomyopathy | 29 | 88 | 1.61e-07 |
| Hepatitis C | 36 | 127 | 3.15e-07 |
| PPAR signaling pathway | 26 | 76 | 3.30e-07 |
| Acute myeloid leukemia | 21 | 55 | 6.78e-07 |
| Long-term potentiation | 24 | 69 | 6.95e-07 |
| Gap junction | 28 | 89 | 7.30e-07 |
| Glycerophospholipid metabolism | 26 | 79 | 7.30e-07 |
| Ribosome biogenesis in eukaryotes | 27 | 84 | 7.30e-07 |
| Amino sugar and nucleotide sugar metabolism | 19 | 47 | 8.27e-07 |
| Toxoplasmosis | 35 | 130 | 1.60e-06 |
| One carbon pool by folate | 11 | 18 | 1.74e-06 |
| Tight junction | 35 | 131 | 1.85e-06 |
| T cell receptor signaling pathway | 31 | 109 | 1.85e-06 |
| Bladder cancer | 16 | 37 | 2.31e-06 |
| Endometrial cancer | 19 | 50 | 2.33e-06 |
| Renal cell carcinoma | 23 | 69 | 2.44e-06 |
| Biosynthesis of unsaturated fatty acids | 13 | 26 | 3.08e-06 |
| Pentose phosphate pathway | 13 | 26 | 3.08e-06 |
| Homologous recombination | 13 | 26 | 3.08e-06 |
| mTOR signaling pathway | 19 | 51 | 3.08e-06 |
| Chemokine signaling pathway | 42 | 178 | 4.78e-06 |
| Rheumatoid arthritis | 25 | 82 | 4.87e-06 |
| Arginine and proline metabolism | 19 | 53 | 5.77e-06 |
| Jak-STAT signaling pathway | 36 | 145 | 7.12e-06 |
| Fructose and mannose metabolism | 15 | 36 | 7.68e-06 |
| Adipocytokine signaling pathway | 22 | 69 | 8.44e-06 |
| Other types of O-glycan biosynthesis | 16 | 41 | 9.75e-06 |
| Calcium signaling pathway | 42 | 185 | 1.24e-05 |
| Vascular smooth muscle contraction | 31 | 120 | 1.35e-05 |
| Aminoacyl-tRNA biosynthesis | 20 | 61 | 1.36e-05 |
| Sphingolipid metabolism | 16 | 42 | 1.36e-05 |
| Huntington's disease | 47 | 218 | 1.45e-05 |
| Oxidative phosphorylation | 37 | 156 | 1.45e-05 |
| Lysine degradation | 17 | 47 | 1.49e-05 |
| Pancreatic secretion | 28 | 104 | 1.51e-05 |
| Protein digestion and absorption | 23 | 78 | 1.94e-05 |
| Steroid biosynthesis | 10 | 19 | 2.50e-05 |
| Basal transcription factors | 14 | 35 | 2.50e-05 |
| Gastric acid secretion | 22 | 74 | 2.54e-05 |
| Colorectal cancer | 21 | 69 | 2.66e-05 |
| Osteoclast differentiation | 30 | 119 | 2.78e-05 |
| Peroxisome | 23 | 80 | 2.87e-05 |
| Phosphatidylinositol signaling system | 22 | 76 | 3.88e-05 |
| Adherens junction | 21 | 71 | 4.13e-05 |
| Parkinson's disease | 37 | 164 | 4.14e-05 |
| Proximal tubule bicarbonate reclamation | 10 | 20 | 4.14e-05 |
| Nicotinate and nicotinamide metabolism | 11 | 24 | 4.35e-05 |
| Salivary secretion | 22 | 78 | 5.74e-05 |
| Circadian rhythm - mammal | 10 | 21 | 6.72e-05 |
| Histidine metabolism | 11 | 25 | 6.72e-05 |
| Pancreatic cancer | 20 | 68 | 6.72e-05 |
| Cell adhesion molecules | 35 | 156 | 7.30e-05 |
| Propanoate metabolism | 13 | 34 | 7.69e-05 |
| Leukocyte transendothelial migration | 28 | 114 | 7.74e-05 |
| RIG-I-like receptor signaling pathway | 19 | 64 | 8.60e-05 |
| Aldosterone-regulated sodium reabsorption | 15 | 44 | 9.14e-05 |
| Base excision repair | 15 | 44 | 9.14e-05 |
| Glycerolipid metabolism | 16 | 49 | 9.28e-05 |
| Melanogenesis | 25 | 98 | 9.63e-05 |
